# Macrophage Migration Inhibitory Factor: A Multifunctional Cytokine in Rheumatic Diseases

**DOI:** 10.1155/2010/106202

**Published:** 2010-12-26

**Authors:** Tsuyoshi Kasama, Kumiko Ohtsuka, Michihito Sato, Ryo Takahashi, Kuninobu Wakabayashi, Kazuo Kobayashi

**Affiliations:** ^1^Division of Rheumatology, Department of Medicine, School of Medicine, Showa University, Tokyo 142-8666, Japan; ^2^Department of Immunology, National Institute of Infectious Diseases, Tokyo 162-8640, Japan

## Abstract

Macrophage migration inhibitory factor (MIF) was originally identified in the culture medium of activated T lymphocytes as a soluble factor that inhibited the random migration of macrophages. MIF is now recognized to be a multipotent cytokine involved in the regulation of immune and inflammatory responses. Moreover, the pivotal nature of its involvement highlights the importance of MIF to the pathogenesis of various inflammatory disorders and suggests that blocking MIF may be a useful therapeutic strategy for treating these diseases. This paper discusses the function and expressional regulation of MIF in several rheumatic diseases and related conditions.

## 1. Introduction

An important advance in our understanding of the pathogenesis of rheumatic diseases such as rheumatoid arthritis (RA), systemic lupus erythematosus (SLE), and systemic vasculitis has been the discovery of the associated abnormal expression and orchestration of several cytokines and inflammatory mediators. Emerging evidence indicates that many of these molecules play key roles during cell activation and contribute to the pathogenesis of disease. For example, the development of the inflammatory pannus, which may be the result of an overproliferation of synoviocytes and infiltration by inflammatory and immune cells, and with subsequent tissue destruction is the histological hallmark of RA [1, 2]. Numerous mediators, including inflammatory cytokines and adhesion molecules, have been implicated in this process [3–6], and it is well known that the orchestration of complicated cytokine networks plays a pivotal role during the evolution of synovitis. Among the cytokines involved is macrophage migration inhibitory factor (MIF), which appears to be an important mediator of inflammatory responses following its secretion from T lymphocytes, macrophages, endothelial cells (ECs), and other inflammatory cells. In this paper, we will focus on the function and expressional regulation of MIF in several rheumatic diseases and related conditions.

## 2. MIF: Overview

Originally identified in the culture medium of activated T lymphocytes as a soluble factor that inhibited macrophage migration [7], MIF is a highly conserved 12.5-kDa protein that exhibits a unique combination of hormone-like, cytokine-like, and thioredoxin-like properties and is now recognized to be a multipotential cytokine involved in the regulation of immune and inflammatory responses [8]. A variety of cell populations have been shown to express and secrete MIF, including T lymphocytes [9], macrophages/monocytes [10], endothelial cells (ECs) [11], eosinophils [12], polymorphonuclear neutrophils (PMNs) [13], epithelial cells [14], smooth muscle cells [15], synovial fibroblasts [16], and anterior pituitary cells [17], which suggests that MIF is involved in a wide array of physiological and pathophysiological processes. As will be described later in detail, the pleiotropic nature of this cytokine is illustrated by the numerous mechanisms implicated in its effects, including activation of mitogen-activated protein kinase (MAPK) signaling [18], upregulated of proinflammatory mediators [8], counterregulation of endogenous glucocorticoids [19, 20] and inhibition of apoptosis [21], among others.

## 3. Induction of MIF

The proinflammatory molecules TNF-*α*, IL-5, IFN-*γ*, transforming growth factor *β*, and lipopolysaccharide (LPS) have all been shown to stimulate MIF mRNA expression and protein secretion [8, 12, 22–24]. It also has been shown that the complement-activated product C5a promotes MIF release from PMNs in vitro and during sepsis [25]. Toll-like receptor 4 (TLR4) stimulation is known to induce the MIF secretion [10] and, intriguingly, TLR2 and TLR4 are both highly expressed in the synovial tissue of RA patients [26]. Moreover, stimulation of dendritic cells (DC) from RA patients with TLR4 ligands elicited higher levels of MIF production than stimulation of immature DC [27]. Finally, Paiva et al. recently showed that macrophages produce MIF upon recognition of immune complex, and the secreted MIF acts as an autocrine/paracrine enhancer of TNF production [28].

## 4. A MIF Receptor

The signal transduction pathways utilized by MIF during its activation of cells and cellular processes are incompletely defined, but one MIF receptor is known to be CD74, the cell surface form of the class II invariant chain [29]. The interaction of MIF with CD74 has been confirmed in “pulldown” experiments, and confocal microscopic examination showed the two proteins to be colocalized within cells [29]. MIF-induced cellular activation appears to be mediated via MAPK and a transcription factor, activator protein 1 (AP-1); that is, MIF appears to signal via classical receptor-dependent activation of MAPK upon binding to CD74 [29]. In addition, recent studies have identified recruitment of transmembrane CD44 as a potential accessory protein required for MIF-CD74 signal transduction [30, 31]. These data show that the serine phosphorylation of the CD74 intracytoplasmic domain by MIF stimulation is dependent upon CD44. Of interest, more recently, crucial roles of chemokine receptors in MIF-CD74 pathway were elucidated. Bernhagen et al. have shown that the chemokine receptors CXCR2 and CXCR4 are functional receptors for MIF [32]. MIF triggered G_*α*i_- and integrin-dependent arrest and chemotaxis of monocytes and T cells, rapid integrin activation, and calcium influx through CXCR2 or CXCR4. Also, CXCR2 and CD74 formed a receptor complex, and monocyte arrest elicited by MIF in inflamed or atherosclerotic arteries involved both CXCR2 and CD74 [32]. Recent advances in understanding of MIF signaling pathway may have an important contribution for new therapeutic strategies for inflammatory/immune diseases. It has been demonstrated that an orally bioavailable MIF antagonist, (S,R)-3-(4-hydroxyphenyl)-4,5-dihydro-5-isoxazole acetic acid methyl ester (ISO-1) [33], inhibited the interaction between MIF and CD74 and reduced functional and histological indices of glomerulonephritis, CD74(+) and CXCR4(+) leukocyte recruitment, and proinflammatory cytokine and chemokine expression in the NZB/NZW F1 and the MRL/lpr mouse strains, two distinct models of SLE [34].

## 5. The Interaction of MIF and Glucocorticoids

MIF is one of cytokine known to be upregulated by glucocorticoids (GCs), which suggests MIF plays a key role in regulating host global responses to infections, as GCs are released from the hypothalamus-pituitary-adrenal axis [17, 35]. GCs elicit MIF secretion both *in vitro *and *in vivo*, but the manner in which MIF is regulated by GC appears to be complex. Although very low concentrations of dexamethasone (10^−14^ M) stimulate MIF secretion [19, 36], this induction is not accompanied by changes in MIF gene expression, and posttranslational mechanisms have been proposed [37]. On the other hand, Leech et al. showed that mRNA expression by RA fibroblast-like synoviocytes (FLS) was induced by the stimulation of lower concentrations of dexamethasone (10^−10^−10^−12^ M) [16]. Upon release, MIF exerts an inhibitory effect on GC activity. For example, recombinant MIF counteracts GC-induced suppression of cytokine production in macrophages and T lymphocytes [9, 19]. *In vivo*, MIF overcomes the protective effect of GCs in murine models of endotoxic shock [19]. And in an antigen-induced arthritis model, exogenous MIF reverses the inhibitory effect of GC on arthritis inflammation, but does not affect GC-induced inhibition of delayed-type hypersensitivity, which suggests there are differences in the sensitivity of inflammatory processes to MIF [38].

## 6. Biological Activities of MIF

### 6.1. Chemotactic Activities

Although MIF was first identified as an inhibitor of macrophage migration [39], induction of leukocyte rolling, adhesion, and transmigration by LPS and other inflammatory mediators is diminished in MIF^−/−^ mice [40]. Similarly, blockade or depletion of MIF reduces leukocyte accumulation in models of infection/endotoxemia, arthritis and atherogenesis [41–43]. Nonetheless, MIF clearly induces adhesion and migration of monocyte-lineage cells in postcapillary venules, and that function is mediated by the chemokine CCL2 (MCP-1), which is induced in ECs by MIF, itself [44]. In addition, expression and secretion of MIF by vascular smooth muscle cells (VSMCs) is increased when the cells are stimulated with oxidized low-density lipoprotein, and recombinant MIF enhances the migration of VSMCs [45]. This suggests that MIF acts in an autocrine and paracrine fashion to modulate the migration of VSMCs, and may be associated with the development of advanced lesions during the course of atherogenesis. MIF also induces chemotaxis in fibroblasts [46] and ECs [47], and the effects of neutralizing MIF in experimental autoimmune myocarditis indicates that it also stimulates migration T cells [48]. In addition, lipopolysaccharide-induced leukocyte-EC interaction was promoted by endogenous MIF, via endogenous GC-independent mechanisms [49]. More recently, Cheng et al. have shown that endogenous MIF promotes leukocyte recruitment via effects on endothelial expression of several adhesion molecules, including E-selectin, intercellular adhesion molecule (ICAM-1), vascular cell adhesion molecule (VCAM)-1, and chemokines, including IL-8 (CXCL8) and CCL2 whereas exogenous MIF facilitates leukocyte recruitment induced by TNF by promoting endothelial P-selectin expression, which contributed to leukocyte rolling [50]. Thus MIF appears to have broad effects on the recruitment of leukocytes, VSMCs, fibroblasts, and leukocyte-EC interactions mediated by several inflammatory molecules in inflammatory/noninflammatory disorders.

### 6.2. Induction of Inflammatory Cytokines by MIF

MIF stimulates release of the proinflammatory cytokines TNF-*α*, IL-1, IL-6, IL-8, and IL-12 from macrophages and up-regulates matrix metalloproteinase (MMP) -1, MMP-3, MMP-9 and MMP-13 in RA FLS [16, 51–57]. In addition, MIF up-regulates the adhesion molecules VCAM-1 and ICAM-1 on ECs and monocytes [58, 59]. 

*In vivo*, MIF deficiency or neutralization has protective effects against lethal bacterial sepsis and septic shock induced by Gram-negative endotoxin [17] or Gram-positive exotoxin [60]. In chronic inflammatory diseases, MIF reduction is associated with lower levels of circulating or local TNF and IL-1 production [61, 62], suggesting MIF is a crucial regulator of inflammatory cytokine expression. *In vitro*, MIF-deficient cells exhibit impaired TNF production in response to LPS, an effect mediated via MIF regulation of TLR4-dependent cellular responses. In different experimental models of sepsis, blocking MIF activity, either through MIF gene disruption or with anti-MIF antibodies, reduces cytokine production by downregulating TLR4 expression [63]. MIF up-regulates macrophage TLR4 expression via the transcription factor PU.1 [63].

In contrast to other proinflammatory cytokines, MIF does not induce nuclear translocation of nuclear factor- (NF-)*κ*B p50 or p60 proteins at concentrations that activate ERK, and inhibitors of the NF-*κ*B pathway do not inhibit MIF-induced biological effects on FLS [64]. In addition, a recent study using an experimental diabetes model showed that MIF^−/−^mice are less susceptible to disease induction, and the reduced susceptibility was associated with lower levels of lymphocyte proliferation and adhesion and decreased splenic production of IL-23 [65]. Because IL-23 can enhance IL-17 production [66, 67], it may be that MIF regulates IL-17 indirectly via its effects on IL-23, which is likely involved in RA and other inflammatory disorders.

### 6.3. Other Activities of MIF

It was recently confirmed that MIF potently stimulates nitric oxide production, which can directly mediate cell injury [68, 69] and enhance macrophage activities such as intracellular killing, phagocytosis, and H_2_O_2_ production [70]. MIF also acts as a potent angiogenic factor in autoimmune diseases [71], exerting its angiogenic effects through induction of such angiogenic mediators as vascular endothelial growth factor (VEGF) [72, 73].

## 7. MIF Expression and Function under Immune/Inflammatory Conditions

### 7.1. MIF in RA

MIF has been implicated in a number of inflammatory and immune-mediated diseases, including RA [16] and other inflammatory arthritis. MIF levels are increased in synovial fluid and synovial tissue from RA patients and patients with juvenile idiopathic arthritis [74–76]. Of interest, synovial MIF immunostaining correlated strongly with disease activity, and reductions in clinical disease parameters were accompanied by significant reductions in synovial MIF [74]. Also, Kim et al. have shown that serum inflammatory markers such as ESR and CRP were correlated with SF levels of MIF, and the SF levels of MIF were found to be higher in patients with bony erosion than in those without. In addition, MIF levels correlated with VEGF levels in both sera and synovial fluids of patients with RA [72]. MIF activates RA FLSs to produce IL-8, cyclooxygenase 2 (COX-2), MMP-1, and MMP-3 via tyrosine kinase-, protein kinase C-, and AP-1-dependent pathways, which contribute to inflammation and tissue destruction [52, 57]. Also, in another function of MIF in synovial inflammation, the effects of MIF on FLS activation and proliferation are dependent on extracellular signal-regulated kinase (ERK) and mitogen-activated protein (MAP) kinase [64, 74]. 

Moreover, in animal models for inflammatory arthritis such as adjuvant-induced arthritis, collagen-induced arthritis, and antigen-induced arthritis (AIA), it was clearly shown that MIF was involved in the pathogenesis of inflammatory arthritis, and the development and severity of the arthritis and the infiltration of inflammatory cells into joint tissues were significantly suppressed by administration of an anti-MIF polyclonal antibody [20, 38, 57, 77], suggesting that MIF may be functionally active during the development of arthritis. Furthermore, exogenous MIF inhibited p53 expression in RA FLS and also increased p53 protein was detected in cells and synovial tissue derived from MIF^−/−^ mice with AIA, suggesting that MIF exerts an antiapoptotic effect in association with its inhibition of p53 in arthritic joints [78]. Finally, recent finding also indicates that MIF may be one of crucial target against anti-TNF therapy in patients with RA [79]. Taken together, these observations indicate that MIF may play important roles in the evolution of the synovitis and joint destruction in RA via modulation of inflammation, angiogenesis and chemotaxis of inflammatory cells.

Although FLS and synovial macrophages are an important cellular source of MIF secretion in synovial tissues of RA [16], MIF was also secreted by dendritic cells (DC) in patients with RA [27]. In monocyte-derived dendritic cells (DC) from RA patients, TLRs significantly enhance production of proinflammatory mediators, including MIF, thereby amplifying the proinflammatory loop seen in arthritis. Moreover, stimulation of DC with TLR4 ligands elicited higher levels of MIF production than stimulation of immature DC from healthy controls or RA patients [27]. Also TLR4 stimulation is known to induce the MIF secretion [10]. Intriguingly, TLR2 and TLR4 are both highly expressed in the synovial tissue of RA patients, and TLR4 ligands are abundant in the serum and synovial fluid of RA patients, suggesting TLR signaling likely occurs in the synovial compartment of these patients [26]. 

The MIF gene is located on chromosome 22q11.2 [80]. The identification of a single-nucleotide polymorphism at position -173 (MIF-173C allele) and a CATT_5–8_ tetranucleotide repeat element in the promoter region of the MIF gene has sparked research into the role of these variants in inflammatory conditions. It has also been demonstrated that the presence of specific alleles of the MIF CATT tetranucleotide repeat correlates with the severity of RA [81]. In addition, Barton et al. found that the MIF-173C allele and the MIF CATT repeat are associated with susceptibility to inflammatory arthritis, but they were unable to find a correlation with disease severity [82]. Martinez showed that the -173C allele in the MIF promoter region is associated with an increased predisposition toward RA, mainly in early-onset patients [83]. The fact that a substantial amount of evidence points toward a role for MIF in the pathogenesis of RA prompted investigation of the potential association between the two MIF genetic variants and the susceptibility to and severity of RA, using a large cohort of well-documented, prospectively followed up patients with RA. Radstake et al. showed that the MIF polymorphisms (-173C and CATT alleles) are associated with the rate of radiologic joint damage, but not with RA susceptibility [84]. Increased MIF levels were shown to correlate strongly with radiologic joint damage, and carriership of the MIF-173C allele or MIF CATT allele was associated with markedly higher levels of circulating plasma MIF, suggesting that MIF expression is genetically determined and can be used as a novel prognostic tool in RA [84]. Consistent with that idea, circulating MIF levels were increased in individuals with juvenile idiopathic arthritis carrying the MIF-173C functional variant, and increased susceptibility to the disease was associated with carriership of either the MIF-173C or CATT allele [75, 76]. It was therefore suggested that for juvenile idiopathic arthritis, the MIF-173C allele is a predictor of poor outcome [75].

### 7.2. MIF in SLE

SLE is an autoimmune disease characterized by multiorgan damage with infiltration of inflammatory cells/immune cells, and the production of autoantibodies. Although the pathogenesis of SLE has not been fully elucidated, recent progress has provided evidence that several cytokines/chemokines are detectable in the sera and also damaged organ of SLE patients during active disease. 

Besides RA, expression and function of MIF were clearly demonstrated in SLE and related conditions. Foote et al. have shown that serum MIF concentrations were positively associated with SLE disease damage. Also, serum MIF was positively associated with current corticosteroid dose and negatively associated with serum creatinine concentration [85]. A marked increase in both glomerular and tubular MIF expression was seen in lupus nephritis, and also in focal segmental glomerulosclerosis (FGS) and mesangiocapillary proliferative GN [86]. In immunohistochemical study, the prominent macrophage and T-cell infiltrate showed were largely restricted to areas with marked upregulation of MIF expression, contributing to glomerular hypercellularity, glomerular focal segmental lesions, crescent formation, tubulitis, and granulomatous lesions. In addition, the positive association of functional polymorphisms of MIF (-173C and CATT alleles), and the prevalence of SLE was shown by Sánchez et al. [87]. 

In the lupus-prone mice, it has been demonstrated that renal MIF expression was significantly higher in MRL/lpr mice compared with nondiseased control mice. Also, MRL/lpr mice with MIF^−/−^ exhibited significantly prolonged survival, and reduced renal and skin manifestations. In addition, renal macrophage recruitment and glomerular injury were significantly reduced in MRL/lpr mice MIF^−/−^, in association with reduction in CCL2 (macrophage chemoattractant protein-1) [88]. In addition, antiapoptotic effect was seen in the active lesion of inflammatory arthritis as described above. This function of MIF may be crucial in the development of SLE, because apoptosis and clearance of apoptotic cells/material are considered key processes in the etiology of SLE.

Matsumoto et al. reported that urinary excretion of MIF is increased in patients with focal glomerular sclerosis and that urinary MIF levels, are higher in patients with active glomerular lesions [89]. Immunocytochemical and *in situ *hybridization studies have shown that MIF is produced by local resident glomerular cells [22], and that administration of a neutralizing anti-MIF antibody dramatically suppresses an immunologically induced disease model of rapidly progressive crescentic glomerulonephritis (GN) [61]. In addition, both MIF mRNA and protein were detected in intrinsic renal cells and glomerular ECs and were markedly up-regulated in more severe forms of GN (e.g., crescentic GN) [22, 86, 90]. The urine MIF concentration was increased 3.4-fold in proliferative nephropathies and especially in crescentic GN (4.5-fold), but not nonproliferative nephropathies [91].There was a significant correlation between the urine MIF concentration and renal MIF expression, but not with serum MIF, indicating a renal origin for the excreted urine MIF. The urine MIF concentration also correlated with the degree of renal dysfunction, histologic damage, and leukocytic infiltration. Mesangial and tubular epithelial cells, as well as glomerular capillary ECs, are the major sources of MIF in GN [22, 90]. The secreted MIF then promotes macrophage activation and secretion of macrophage-derived cytokines, including IL-1 and fibroblast growth factor, which may induce mesangial cell proliferation [10, 92]. It thus appears that when combined with other factors, MIF expressed in the inflamed kidney contributes to the development of the renal damage in GN.

### 7.3. MIF in Systemic Vasculitis

We recently showed that serum MIF levels are significantly increased in some patients with systemic vasculitis [94]. Notably, significantly elevated levels of serum MIF were seen in patients with microscopic polyangiitis (MPA), which is a small vessel vasculitis, but not in patients with medium vessel vasculitis, such as polyarteritis nodosa, or large vessel vasculitis, such as giant cell arteritis and Takayasu arteritis. The elevated MIF levels seen in MPA patients correlated positively with indexes of disease activity, including Birmingham vasculitis activity scores, CRP levels and ESR. Furthermore, MIF levels were significantly diminished in MPA patients exhibiting clinical improvement after treatment. Similarly, serum MIF levels were elevated in patients with antineutrophil cytoplasmic antibody- (ANCA-) related vasculitis [95], as well as in Wegener's granulomatous and Kawasaki disease [96]. These findings indicate that MIF expression is not specific to RA, but may also function as an important regulator of systemic vasculitis. Serum levels of endothelium-related molecules such as adhesion molecules and EC-derived cytokines are reportedly increased in patients with vasculitis [102, 103]. Indeed, vasculitis affecting small vessels may be associated with dysregulated EC function [104]. In patients with MPA, for example, the origin of the elevated serum MIF appears to be ECs and/or inflammatory cells such as monocytes and PMNs [10, 25]. Once secreted, MIF likely participates in regulating EC proliferation [47]. There is also a positive correlation between serum MIF levels and MPO-ANCA titers in MPA patients [94]. Although there are no data on the capacity of MPO-ANCA to stimulate secretion of any cytokine, including MIF, we would expect it to be related to disease activity and MIF levels, because there appears to be positive relation between MPO-ANCA titers and vasculitis disease activity [105]. As mentioned earlier, MIF up-regulates ICAM-1 on ECs [58], as well as the expression and secretion of other inflammatory cytokines, including TNF-*α* and IL-8 [16, 57]. This in turn would be expected to enhance recruitment of leukocytes to sites of inflammation, which involves adhesion molecule-dependent interactions with ECs. 

We also recently observed that serum MIF levels are significantly higher in RA patients with vasculitis (rheumatoid vasculitis; RV) than in those without it (manuscript in preparation). We found that in RV patients, there are significant positive correlations between MIF levels and vasculitis disease activity scores and serum levels of immune complex and a significant negative correlation between MIF levels and serum complement levels. Notably, MIF levels in RV patients also correlated significantly with levels of thrombomodulin, expression of which is associated with endothelial damage and/or vascular inflammation. Collectively, these findings suggest dysregulated orchestration of the activities of MIF, adhesion molecules, and cytokines expressed by ECs and/or leukocytes plays a crucial role in the development of systemic vasculitis (e.g., MPA and RV).

### 7.4. MIF in Other Rheumatic Diseases and Related Conditions

Serum MIF levels and dermal fibroblast-derived MIF synthesis are both up-regulated in scleroderma, suggesting that MIF participates in the amplifying proinflammatory loop that leads to sclerodermal tissue remodeling [97]. Serum MIF levels are also significantly elevated in patients with primary Sjögren's syndrome, especially in those with increased *γ*-globulins [98]. It has been shown that MIF signaling stimulates B cell proliferation [106], and that neutralization of MIF significantly inhibits antibody production *in vivo* [9]. Increased production of MIF might therefore contribute to hypergammaglobulinemia and possibly reflect the disease activity of Sjögren's syndrome. Ankylosing spondylitis (AS) is a chronic inflammatory disease mainly affecting the spine and sacroiliac joints. MIF levels were significantly higher in the AS patients than in normal individuals, which correlated with the Bath Ankylosing Spondylitis Metrology Index a composite clinical index for AS [99]. Furthermore, important pathogenic contributions of MIF have been suggested by studies in adult-onset Still's disease [100], ocular inflammation [107], relapsing polychondritis [101], experimental autoimmune encephalomyelitis, a model of multiple sclerosis [71], inflammatory bowel disease, Crohn's disease, and experimental colitis [54, 93].

## 8. Conclusion

**Figure 1:**
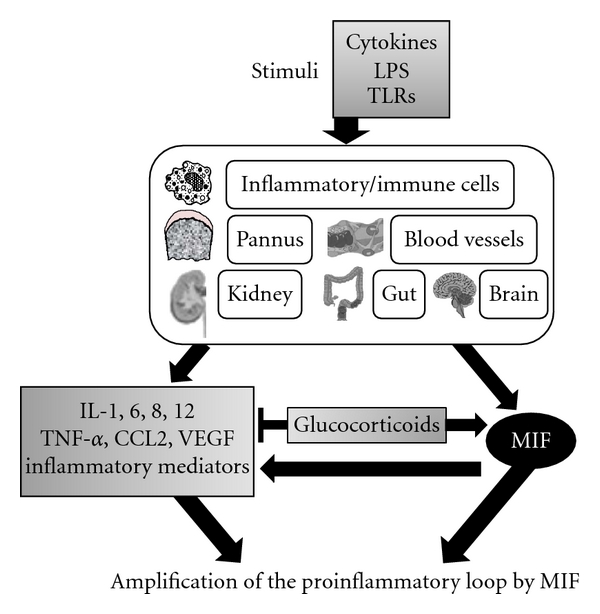
The orchestration of complicated cytokine networks by MIF in inflammatory/immune responses. MIF is a multipotent cytokine involved in the regulation of immune and inflammatory responses via other various mediators, and plays a key role in regulating a number of inflammatory and immune-mediated diseases seen in pannus, blood vessels, kidney, gut, and brain.

The biological activities of MIF and its relevance in various diseases are summarized in Tables 1 and 2. The central involvement of this multifunctional cytokine highlights its importance in the pathogenesis of inflammatory diseases (Figure 1). Moreover, it suggests that blocking MIF may be a useful therapeutic strategy for treating these diseases.

## Figures and Tables

**Table 1:** Biological activities of MIF.

Chemotactic activities	
Monocytes	stimulation/inhibition: dependent on its concentration
T lymphocytes	
ECs	
Vascular smooth muscle cells	
Fibroblasts	
Angiogenic activities	
Antiapoptotic activities	
Stimulation of cell proliferation	
Induction of mediators	
Cytokines	TNF-*α*
	IL-1, 6, 8, 12
	CCL2
Growth factor	VEGF
Adhesion molecules	
	ICAM-1
	VCAM-1
	E-selectin
	P-selectin
Proteinases	MMP-1, 3, 9, 13
Nitric oxide	
Superoxide	

Chemotactic activities of MIF against monocytes/macrophages may be dependent on its concentration. VEGF; vascular endothelial growth factor, ICAM-1; intercellular cell adhesion molecule-1, VCAM-1; vascular cell adhesion molecule-1, MMP; matrix metalloproteinase.

**Table 2:** Involvement of MIF in various pathological conditions

Diseases	References
RA, including rheumatoid vasculitis	[16, 27, 52, 57, 72, 74, 79]
Crohn's disease	[54, 93]
Juvenile idiopathic arthritis	[75, 76]
Systemic lupus erythematosus	[85–87]
Crescentic glomerulonephritis	[86, 91]
Focal glomerular sclerosis	[89]
Microscopic polyangiitis	[94, 95]
Wegener's granulomas	[95]
Kawasaki disease	[96]
Systemic sclerosis	[97]
Sjögren's syndrome	[98]
Ankylosing spondylitis	[99]
Adult-onset Still's disease	[100]
Relapsing polychondritis	[101]
